# Degeneration pattern in somatic embryos of *Pinus sylvestris* L.

**DOI:** 10.1007/s11627-016-9797-y

**Published:** 2017-01-26

**Authors:** Malin Abrahamsson, Silvia Valladares, Irene Merino, Emma Larsson, Sara von Arnold

**Affiliations:** 1 Department of Plant Biology, Uppsala BioCenter, Swedish University of Agricultural Sciences, P.O. Box 7080, 750 07 Uppsala, Sweden; 2 Plant Biotechnology Laboratory, Fundación Promiva, Ctra M501 Km 5,1, 28670 Villaviciosa de Odón, Madrid Spain

**Keywords:** Embryo degeneration, Embryo patterning, Initiation of embryogenic culture, *Pinus sylvestris*, Scots pine, Somatic embryogenesis

## Abstract

Somatic embryos can be used for propagating forest trees vegetatively, which is of great importance for capturing the genetic gain in breeding programs. However, many economically important *Pinus* species are difficult or impossible to propagate via somatic embryogenesis. In order to get a better understanding of the difficulties to propagate *Pinus* species via somatic embryogenesis, we are studying the developmental pathway of somatic embryos in different cell lines. In a previous study, we showed that the morphology of early somatic embryos in Scots pine (*Pinus sylvestris*) differs between cell lines giving rise to normal or abnormal cotyledonary embryos. In this study, we have compared the proliferation and degeneration pattern of early and late embryos in a normal and abnormal cell line. In both cell lines, a high frequency of the embryos degenerated. Among the degenerating embryos, two main degeneration patterns could be distinguished. In the normal cell line, the embryos degenerated similar to how the subordinate embryos are degraded in the seed. In the abnormal cell line, the degeneration of the embryos resulted in a continuous loop of embryo degeneration and differentiation of new embryos. We observed a similar degeneration pattern when embryogenic tissue was initiated from megagametophytes containing zygotic embryos at the stage of cleavage polyembryony. Based on our results, we suggest that the degeneration pattern in abnormal cell lines starts during initiation of embryogenic cultures.

**Electronic supplementary material:**

The online version of this article (doi:10.1007/s11627-016-9797-y) contains supplementary material, which is available to authorized users.

## Introduction

Vegetative propagation of the economically important conifers makes it possible to capture the genetic gain obtained in the breeding program. Thus, propagation by somatic embryogenesis has great potential for application in forestry. However, for many *Pinus* species, several problems remain to be solved before somatic embryogenesis can be used as a reliable propagation tool (Klimaszewska *et al.* 2007; Bonga *et al.* 2010).

In most *Pinus* species, embryogenic cultures are initiated from immature zygotic embryos (Klimazewska *et al.* 2007, with ref.). Generally, in these species, the apical cells in the zygotic proembryo cleave and develop into four separate embryos. Owing to the cleavage of the terminal proembryo units, the process is termed cleavage polyembryony (Buchholz 1918). Since the four embryos are derived from the same zygote, they are genetically identical. Initially, the four embryos are equal in size, but over time, one embryo becomes dominant and develops further to a mature cotyledonary embryo (Dogra 1967). The remaining embryos, termed subordinate embryos, are degraded by programmed cell death (PCD) (Filonova *et al.* 2002). When using immature zygotic embryos at the stage of cleavage polyembryony as primary explants, the embryogenic tissue might be initiated by a continuation of the cleavage process (Bozhkov *et al.* 1997; Park *et al.* 2006). Polyembryony can be a potential problem in somatic embryogenesis in *Pinus* (Klimaszewska *et al.* 2007).

The initiation frequency of embryogenic tissue is under genetic control (Park *et al.* 1993; MacKay *et al.* 2006), and for many *Pinus* species, it has only been possible to establish embryogenic cultures, from which normal cotyledonary embryos can be regenerated, from a limited number of genotypes. The problems emerging during maturation later result in very low germination frequency and reduced vigor of somatic embryo plants (Klimaszewska *et al.* 2007, with ref.). The protocols for propagating *Pinus* via somatic embryos have successively been improved by adjusting the culture conditions, including different basal media and the types and concentrations of plant growth regulators (PGRs) (Klimazewska *et al.* 2007, with ref.). Although advances have been obtained in initiation and maturation, further studies are required in order to control the development of somatic embryos in *Pinus*.

We have previously analyzed the development of somatic embryos in different cell lines of Scots pine (*Pinus sylvestris*), including cell lines from which normal cotyledonary embryos develop, as well as cell lines giving rise to abnormal cotyledonary embryos (Abrahamsson *et al.* 2012). We observed that the frequency of cells undergoing PCD was high in both types of cell lines and that the formation of supernumerary suspensor cells during differentiation of early embryos was more common in the abnormal cell line. The aim of this work was to thoroughly compare the proliferation and differentiation pattern of somatic embryos from two contrasting cell lines developing normal and abnormal cotyledonary embryos. A high frequency of early and late embryos degenerated in both cell lines, although the degeneration pattern differed for embryos from the two cell lines. In the normal cell line, the degenerated embryos were degraded, while in the abnormal cell line, the degenerated embryos started to differentiate new abnormal early embryos. This is the first report that describes degeneration patterns of somatic embryos in Scots pine, and the results suggest that when embryogenic cultures are established from immature zygotic embryos, there is a risk that the cell lines will be abnormal and give rise to abnormal cotyledonary embryos.

## Material and methods

### Plant material

Two embryogenic cell lines of Scots pine (*P. sylvestris* L.) have been used in this study: cell line 12:12 which produces mainly normal cotyledonary embryos and cell line 3:10 which gives rise to abnormal cotyledonary embryos. Both cell lines were initiated in the year 2000 from immature seeds collected from open-pollinated trees growing in a seed orchard in central Sweden (Burg *et al.* 2007). As soon as the cell lines had been established, they were cryopreserved. Samples from the cryopreserved cell lines were, after about 10 yr, thawed from liquid nitrogen and established on solidified medium before starting the experiments. DCR medium (Gupta and Durzan 1985) modified as described previously (Burg *et al.* 2007) was used as the basal medium. The pH was adjusted to 5.8 ± 0.05 prior to autoclaving. Filter-sterilized glutamine was added into an autoclaved cooled medium. The cultures were maintained in liquid or on solidified (0.35% (*w*/*v*) gellan gum, Gelrite^®^, Kelko, Atlanta, GA) proliferation medium containing sucrose (30 mM) and the plant growth regulators (PGRs) 2,4-dichlorophenoxyacetic acid (2,4-D) and benzyladenine (BA) at 9.0 and 4.4 μM, respectively (Abrahamsson *et al.* 2012). To stimulate the differentiation of early embryos and the development of late and cotyledonary embryos, the cultures were first transferred to liquid or solidified pre-maturation medium lacking PGRs for 2 to 3 wk and then to solidified maturation medium, containing 7.5% (*w*/*v*) polyethylene glycol (PEG 4000), 60 μM abscisic acid (ABA), and 83.2 mM maltose. Filter-sterilized ABA was added into an autoclaved cooled medium. The cultures were incubated in darkness at 22 ± 1°C. Embryogenic tissue cultured on solidified medium was sub-cultured every second week. Nine pieces (ca. 50 mg fresh weight) were incubated in each Petri dish (92 mm in diameter). Embryogenic tissue cultured in liquid medium was sub-cultured every wk. Briefly, 5 ml of sedimented cells was transferred to 100 ml fresh medium in 250-ml Erlenmeyer flasks. The cultures were grown on a gyratory shaker (125 rpm).

### Morphological analysis

The development of somatic embryos was separated into ten consecutive stages as described previously (Abrahamsson *et al.* 2012): stage 1a, proliferating embryogenic cell aggregates; stage 1b, slightly globular structures; stage 1c, differentiating early embryos; stage 2, early embryos; stage 3, late embryos; stage 4, late embryos before cotyledon differentiation; stages 5–7, maturing embryos with developing cotyledonary primordia; and stage 8, fully matured cotyledonary embryos.

For characterizing the development of embryos, embryos at stages 2 to 4 were sampled from cell line 12:12 after 4 and 6 wk on maturation medium and from cell line 3:10 after 1, 2, and 3 wk on maturation medium, when early and late embryos had differentiated (Abrahamsson *et al.* 2012). The sampled embryos were transferred to fresh maturation medium. The frequency of embryos at different developmental stages (stages 2–7) was estimated in cell line 12:12 after 4 and 6 wk on maturation medium. Presented data were based on 554 embryos from 12 biological replicates. The degeneration pattern was characterized for embryos (stages 2–4) in both cell lines 12:12 and 3:10. The analyses included 490 embryos from 12 biological replicates from cell line 12:12 and 811 embryos from 15 biological replicates from cell line 3:10. The morphology of the embryos was recorded after 1 and 2 wk. All embryos were examined under a Leica MZFLIII stereomicroscope (Leitz, Stuttgart, Germany) or a Zeiss Axiovert 10 inverted microscope (Zeiss, Oberkochen, Germany). Significant differences in developmental and degeneration pattern were determined by ANOVA (SPSS^®^ v.23 statistical software package).

### Initiation of embryogenic tissue from immature zygotic embryos and from cotyledonary zygotic and somatic embryos

Cones, containing seeds with embryos at the stage of cleavage, were collected randomly from trees in Uppsala, Sweden (59° latitude, 17° longitude). The first collection was made on the 23rd and 27th of June year 2008, and the second collection was made between the 3rd and 13th of July year 2015. The cones were surface sterilized first in 70% (*v*/*v*) ethanol for 1 to 2 min and thereafter in 20% Klorin original (Colgate-Palmolive, New York, NY) supplemented with a few drops of Tween^®^ 20 for 20 min. The cones were then abundantly rinsed with sterile distilled water and placed in sterile empty Petri dishes. The cones were opened, and the immature seeds were isolated. In total, 1530 megagametophytes were excised from the seeds and placed horizontally on the proliferation medium supplemented with 250 mg l^−1^ cefotaxime (Sandoz A/S, Copenhagen, Denmark). Embryogenic tissue started to protrude from a total of 19 megagametophytes after 2 wk. Samples of embryogenic tissue were collected from the earliest protruding tissue and at several stages until proliferating embryogenic tissue was established. The samples were examined under a light microscope (Zeiss Axioplan or Leica DMI 4000).

The effect of treatment with trichostatin A (TSA) on initiation of embryogenic tissue was tested on cotyledonary zygotic embryos as well as on and cotyledonary somatic embryos from cell lines 12:12 and 3:10. TSA (Sigma-Aldrich, St. Louis, MO) was dissolved in DMSO to obtain a stock solution of 10 mM. The cotyledonary embryos were incubated for 4 wk on proliferation medium containing 0, 10, 25, or 50 μM TSA. Thereafter, the embryos were transferred to proliferation medium lacking TSA. Initiation of new embryogenic tissue was estimated after 4 wk on proliferation medium. The presented data for initiation frequencies were based on three biological replicates with each replicate including at least 75 cotyledonary embryos per treatment. The newly initiated embryogenic tissue was isolated and cultured separately on proliferation medium. When new embryogenic sub-lines were established, the developmental pattern of the embryos was analyzed on maturation medium. All cultures were sub-cultured every second wk.

### Histological and histochemical analyses

For histological analyses, embryos from cell line 3:10 were fixed and embedded in Histowax^®^ (Karlgren *et al.* 2009). The embedded embryos were serially sectioned (8 μm) using a Microm HM 350 microtome. Briefly, 200 μl Western Blue^®^ Substrate (Promega, Madison, WI) was added to each slide, incubated at RT for 1 h, washed with PBS, and examined under a light microscope (Zeiss Axioplan).

The cuticle of somatic embryos from cell line 3:10 was stained in freshly prepared Oil Red O (Sigma-Aldrich), 0.3% (*w*/*v*) in water, for 5 min and then washed in water (Akin *et al.* 2004) before being examined under a light microscope (Zeiss Axioplan) in dark field.

The fluorescent nucleic acid stain SYTOX^®^ Orange nucleic acid (Life Technologies, Carlsbad, CA) was used in order to detect dead cells. SYTOX^®^ Orange nucleic acid, which is impermeable to living cells but easily penetrates membranes in dead cells, binds to double-stranded DNA or RNA in cells such as late apoptotic and necrotic cells. The samples were stained with freshly prepared SYTOX^®^ Orange nucleic acid, (1:1000) in water, for 15 min and then examined under a Leica DMI 4000 microscope.

## Results and discussion

### A large proportion of early somatic embryos degenerate

**Fig. 1.**
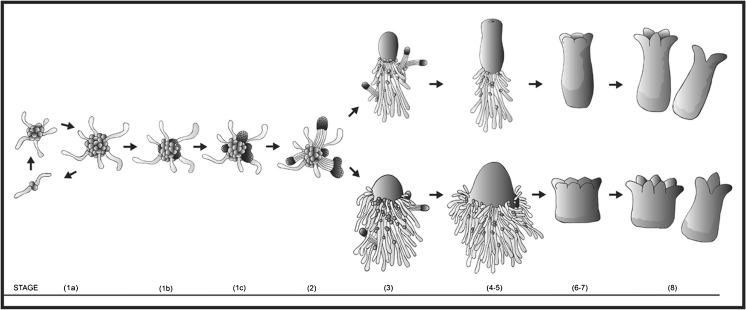
Schematic drawing of the development of somatic embryos from cell line 12:12 giving rise to embryos with normal morphology (*upper row*) and from cell line 3:10 giving rise to embryos with abnormal morphology (*lower row*). The developmental stages are indicated at the *bottom*. Proliferating embryogenic cultures, maintained on proliferation medium, consist of a mixture of small meristematic cells and elongated, vacuolated cells that are clustered into larger aggregates from which smaller aggregates differentiate and disperse (*stage 1a*). When transferred to pre-maturation medium, the aggregates continue to proliferate simultaneously as parts of the aggregates develop into globular structures covered by a smooth surface (*stage 1b*). Early embryos start to differentiate from the globular structures on maturation medium (*stage 1c*). Subsequently, early embryos with a distinct apical-basal axis composed of an embryonal mass in the apical part and a suspensor in the basal part develop (*stage 2*). A high frequency of the early embryos in cell line 12:12 proceed their development into normal late embryos (*stage 3*) and further into normal cotyledonary embryos (*stage 8*). Many of the early embryos in cell line 3:10 develop into abnormal late embryos (*stage 3*) with a cone-shaped embryonal mass and supernumerary suspensor cells and further into cotyledonary embryos with shortened or aborted hypocotyls (*stage 8*). The developmental pathway is based on the original publication by Abrahamsson *et al.* (2012).

We have previously described the general developmental pathway of somatic embryos in cell lines yielding normal cotyledonary embryos and cell lines yielding a large proportion of abnormal cotyledonary embryos in Scots pine (Abrahamsson *et al.* 2012; Fig. 1). To further understand the differences between normal and abnormal cell lines, we thoroughly compared the developmental pathway in the two contrasting cell lines: the normal line 12:12 and the abnormal line 3:10. Early embryos in cell line 3:10 differentiated after 1 to 2 wk on maturation medium, but first after 2 to 4 wk in cell line 12:12. Despite the differences in time, the developmental pattern up to stage 2 was similar in both cell lines.

A high frequency of the stage 2 embryos degenerated (Table 1). However, the proportion of degenerating embryos successively decreased when the embryos had developed further. At the same time, as the degeneration process of early embryos continued on maturation medium, new embryos differentiated and developed further, so that embryos at a wide range of different developmental stages could be observed at the same time point (Table S1).

**Table 1.** Embryo degeneration during maturation treatment

Cell line	Embryo stage	Normal development (%)	Degeneration (%)	
			Degeneration pattern (i)	Degeneration pattern (ii)
12:12	2	22.4 ± 2.9 ^1^	72.8 ± 1.9*a	4.8 ± 0.6 a
	3	49.0 ± 4.7 ^2^	47.5 ± 5.2*b	3.5 ± 1.3 a
	4	83.1 ± 3.9 ^3^	16.9 ± 3.3*c	0.0
3:10	2	28.0 ± 7.4 ^1^	36.9 ± 10.5 a	35.1 ± 12.0 a
	3	55.7 ± 8.8 ^2^	15.5 ± 5.7*b	28.8 ± 5.9 b
	4	63.8 ± 5.5 ^2^	14.0 ± 0.0 b	22.2 ± 5.5 b

Embryos at stages 2, 3, and 4 were sampled from cell line 12:12 after 4 and 6 wk on maturation medium and from cell line 3:10 after 1, 2, and 3 wk on maturation medium. The proportion of normal and degenerating embryos were recorded after 2 wk. The degenerating embryos were classified as type (i) or (ii) as shown in Fig. 3 *c*–*j*. The presented data give the proportion of embryos at each developmental stage showing normal development or a specific degeneration pattern (± SE), based on 12 (12:12) and 15 (3:10) biological replicates, in a total of 490 and 811 embryos, respectively. *Different numbers* indicate significant differences in the frequency of embryos at developmental stages 2, 3, and 4 developing normally within each cell line (*P* ≤ 0.001). *Asterisks* indicate significant differences between the two degeneration patterns at each developmental stage within each cell line (*P* ≤ 0.001). *Different letters* indicate significant differences in the frequency of embryos going through degeneration pattern (i) or (ii) among different developmental stages within each cell line (*P* ≤ 0.001)

**Fig. 2.**
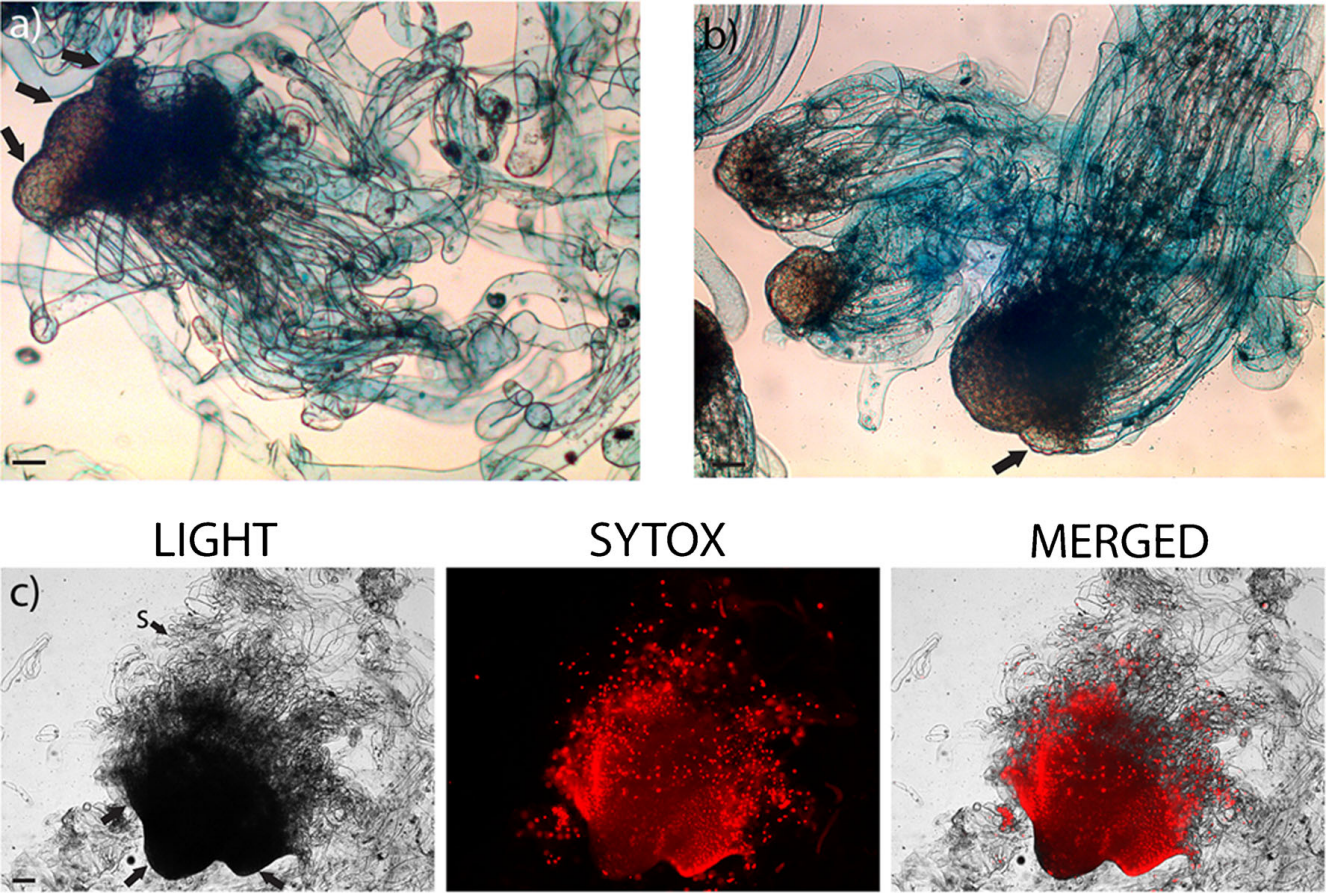
Lobing embryos from cell line 3:10. (*a*) Abnormal stage 3 embryo with three developing lobes, denoted by *arrows*, after 2–5 wk on maturation medium. (*b*) Abnormal stage 2 to 3 embryos after 2–5 wk on maturation medium. Note the developing lobe, denoted by *arrow*, and the presence of supernumerary suspensor cells on one of the embryos. (*c*) An extensive lobing stage 3/4 embryo. The embryo was stained with SYTOX^®^ and observed under a fluorescence microscope. *Red fluorescence* indicates dead cells. The lobes are denoted by *arrows*. The embryos in the figure (*a*, *b*) were stained with Evans blue. *s* suspensor cells. *Bars* 100 μm.

In cell line 12:12, most of stage 3 embryos had a normal morphology and developed into cotyledonary embryos with a normal morphology (Fig. 1, upper row). Contrastingly, in cell line 3:10, many embryos at stage 3 had developed a cone-shaped embryonal mass and differentiated supernumerary suspensor cells (Fig. 1, lower row). The radial growth, which persisted throughout the maturation process, resulted in that many cotyledonary embryos became stunted with shortened or aborted hypocotyl. In cell line 3:10, many embryos at stages 2 to 4 developed lobes (Fig. 2 *a*, *b*). By treating lobing embryos with the nucleic acid stain SYTOX^®^ Orange nucleic acid, we could observe that the lobing embryos often were dying or dead (Fig. 2 *c*).

In zygotic embryos, lobing or partial cleavage is the result from an unbalanced growth rate between different domains in the embryonal mass (Dogra 1967). Furthermore, if the lobes develop their own independent embryonal tube cells, lobing of the embryonal mass could also result in cleavage polyembryony. Extensive lobing is common in zygotic embryos with retarded development and can result in embryo degradation (Singh 1978, with ref.). Thus, our results indicate that somatic embryos in cell line 3:10 develop in a similar way as zygotic embryos with retarded development. The abnormal morphologies observed among cotyledonary somatic embryos of Scots pine have not been reported for zygotic embryos in seeds collected from the central part of Sweden, i.e., the area from where the seeds were collected for establishing the embryogenic cell lines used in this study and previous studies (Burg *et al.* 2007). However, in seeds collected from Scots pine growing in northern parts of Sweden, where the climate is much harsher, a high frequency of abnormal embryos has been found (Dogra 1967). The abnormalities were suggested to be caused by stress-induced persistent polyembryony. If the embryos survived to maturity, they were malformed, often with increased width and lack of apical-basal polarity along the shoot-root axis (Dogra 1967). Thus, our results suggest that somatic embryos in cell lines similar to 3:10 remain at a developmental stage comparable to persistent polyembryony. At present, we do not know the regulatory mechanisms that make one embryo dominant above the others so that it can develop further into a normal cotyledonary embryo.

### Two degeneration patterns can be distinguished during somatic embryo development in Scots pine

**Fig. 3.**
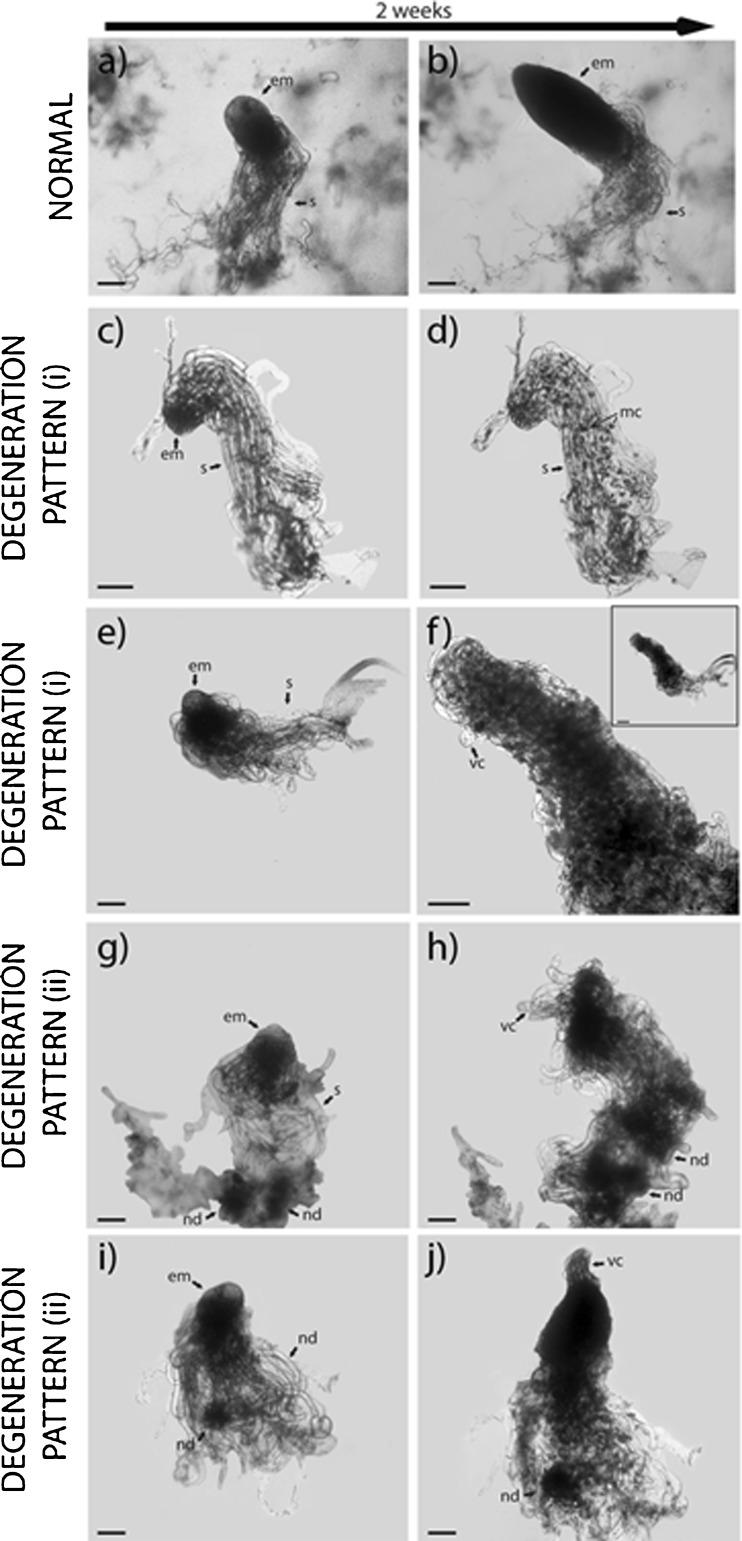
Tracking of somatic embryos with different degeneration patterns. Embryos at stages 2 and 3 were sampled from cell line 12:12 after 4 and 6 wk on maturation medium and from cell line 3:10 after 1, 2, and 3 wk on maturation medium. The sampled embryos were transferred to fresh maturation medium, and their developmental pattern was recorded after 2 wk. The degenerating embryos were classified as type (i) or (ii). (*a*, *b*) Normal development. (*a*) An embryo at stage 3 from cell line 3:10. (*b*) The same embryo as in (*a*), at stage 4, after another 2 wk on maturation medium. (*c*, *d*) Degeneration pattern (i). (*c*) An embryo at stage 2 from cell line 12:12. (*d*) The same embryo as in (*c*) after another 2 wk on maturation medium. Note the disintegration of the embryonal mass. (*e*, *f*) Degeneration pattern (i). (*e*) An embryo at stage 3 from cell line 3:10. (*f*) The same embryo as in (*e*) after another 2 wk on maturation medium. The whole embryo is shown in the inserted picture. Note the disintegration of the embryonal mass and the vacuolated cells in the embryonal mass. (*g*, *h*) Degeneration pattern (ii). (*g*) An embryo at stage 3 from cell line 3:10. (*h*) The same embryo as in (*g*) after another 2 wk on maturation medium. Note the degeneration of the embryonal mass; the differentiation of elongated, vacuolated cells from the embryonal mass; and the presence of meristematic nodule-like structures in the suspensor. (*i*, *j*) Degeneration pattern (ii). (*i*) An embryo at stage 3 from cell line 3:10. (*j*) The same embryo as in (*i*) after another 2 wk on maturation medium. Note the cluster of differentiating elongated, vacuolated cells from the apical part of the embryonal mass and the presence of meristematic nodule-like structures in the suspensor. *em* embryonal mass, *mc* meristematic cells, *nd* nodule-like structures, *s* suspensor cells, *vc* vacuolated cells. *Bars* 100 μm.

**Fig. 4.**
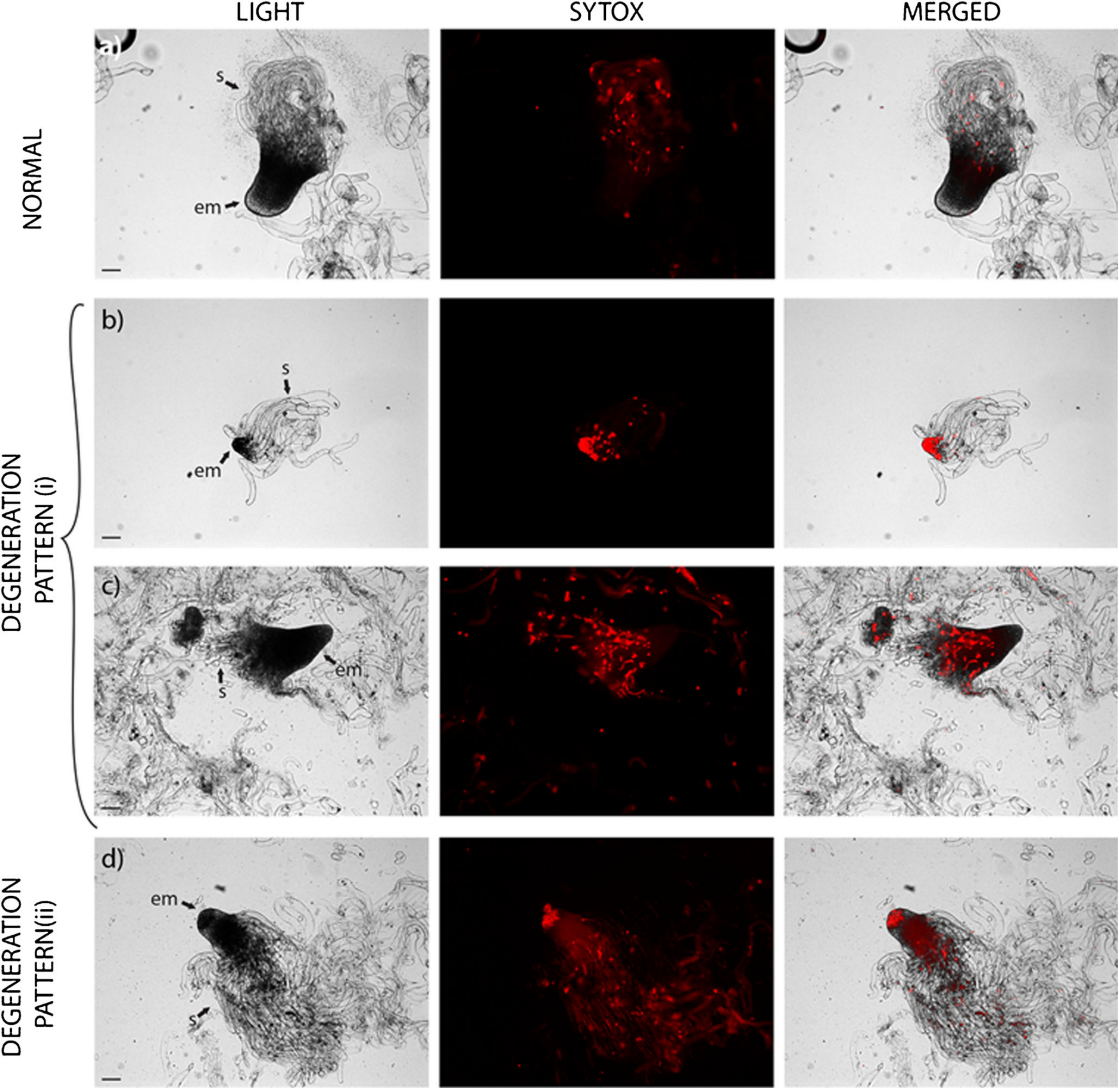
Dead cells in somatic embryos during maturation treatment. Embryos from cell lines 12:12 and 3:10 were selected after 6 wk on maturation medium. The embryos were stained with SYTOX^®^ and observed under a fluorescence microscope. *Red fluorescence* indicates dead cells. (*a*) Normal embryo at stage 3/4 from cell line 12:12 with dead suspensor cells. (*b*) Embryo at stage 2 from cell line 12:12 degenerating according to degeneration pattern (i). Note the dead cells in the embryonal mass. (*c*) Embryo at stage 3 from cell line 12:12 degenerating according to degeneration pattern (i). Note the basal-apical progression of the dead cells in the embryo. (*d*) Embryo at stage 3 from cell line 3:10 degenerating according to degeneration pattern (ii). Note the dead cells in the apical part of the embryonal mass. *em* embryonal mass, *s* suspensor cells. *Bars* 100 μm.

In normally developing embryos, the suspensor cells were gradually degraded while the cells in the embryonal mass remained intact (Figs. 3 *a*, *b* and 4 *a*). Among the degenerating embryos, two main degeneration patterns could be distinguished: (i) the embryonal mass disintegrated (Fig. 3 *c*, *d*) and/or cells in the embryonal mass became vacuolated (Fig. 3 *e*, *f*) or (ii) elongated, vacuolated cells differentiated from the embryonal mass and meristematic nodule-like structures emerged in the suspensor (Fig. 3 *g*–*j*). New embryos differentiated, and subsequently, these embryos degenerated into less organized embryos, causing a continuous loop of embryo degeneration and differentiation of new embryos. We have previously shown that extensive PCD is occurring in embryogenic cultures of Scots pine (Abrahamsson *et al.* 2012). To assess this cell death further and to more thoroughly study the different degeneration patterns, we treated developing embryos with SYTOX^®^, which stains cells undergoing not only PCD but also other types of cell death, such as for example necrosis. In embryos undergoing degeneration pattern (i), many cells in the embryonal mass were dead at stage 2 (Fig. 4 *b*), while at stage 3, the cells at the basal part of the embryonal mass died first and, successively, the cell death process proceeded towards the apical part (Fig. 4 *c*). This basal-apical cell death pattern was highly similar to what has been shown to occur in subordinate embryos degraded by PCD in the seed (Filonova *et al.* 2002). In contrast, in embryos undergoing degeneration pattern (ii), clusters of dead cells were detected in the apical part of the embryonal mass (Fig. 4 *d*). In cell line 12:12, degeneration pattern (i) was significantly dominant at all developmental stages analyzed (Table 1). However, in cell line 3:10, degeneration pattern (ii) was significantly dominant among stage 3 embryos, while both degeneration patterns occurred to the same extent among embryos at stage 2 and stage 4. Together, our observations indicate that in cell lines giving rise to normal cotyledonary embryos, a large proportion of developing embryos degenerate similar to subordinate embryos in the seed, which results in elimination of the degenerated embryo. In contrast, the major degeneration pattern in cell lines giving rise to abnormal cotyledonary embryos causes a continuous loop of embryo degeneration and differentiation of new embryos.

The formation of a protoderm at early stages of embryo development restricts cell expansion and is essential for the functional development of the embryo (Dodeman *et al.* 1997). The embryonal mass in most somatic embryos at developmental stages 2 and 3 in both cell line 12:12 and cell line 3:10 had a smooth surface when observed under the microscope (Abrahamsson *et al.* 2012), indicating that they had differentiated a protoderm. The presence of a protoderm was also confirmed in sectioned embryos (Fig. S1a). Protodermal and epidermal cells secrete lipids to their outer cell wall, resulting in the formation of a cuticle early during embryo development (Yeats and Rose 2013). The abnormalities observed in embryos at stages 2 and 3 could reflect a disturbed cuticularization process. In order to elucidate if a cuticularized layer had been formed, embryos from cell line 3:10 were stained with Oil Red which specifically stains lipids (Akin *et al.* 2004). An intact cuticle could be observed in embryos at stage 2 (Fig. S1b) and in most embryos at stages 3 and 3/4 (Fig. S1c,d). The cuticularized layer was degraded in embryos that degenerated according to pattern (i) (Fig. S1e), but not in the embryos degenerated according to pattern (ii) (Fig. S2f). Our results suggest that the abnormalities observed in embryos at stages 2 and 3 in cell line 3:10 were not a consequence of the malformation of the protoderm, but rather that the protoderm was degraded as a consequence of the degeneration pattern.

In Scots pine, the zygotic embryo develops within the megagametophyte of the seed. Similar to the endosperm in angiosperms (Pagnussat *et al.* 2005), the megagametophyte plays an important role in regulating embryo development. Damage or injury of the megagametophyte can lead to embryo degradation (Dogra 1967) just as abnormal endosperm development can result in embryo abortion (Lopes and Larkins 1993, with ref.). The embryo influences the megagametophyte, so that if the embryo(s) die in the ovule, the megagametophyte will start to degenerate (Sarvas 1962). Somatic embryos are cultivated in the absence of the megagametophyte. However, in the medium conditioned by embryogenic cultures, substances accumulate which have been found to sustain or promote somatic embryogenesis (Dyachok *et al.* 2002, with ref.). Numerous proteins identified in the conditioned medium were seed-specific, and therefore, it has been assumed that some cells in cell cultures have endosperm-megagametophyte-like properties. In order to determine if surrounding proliferating cells in embryogenic cultures could stimulate the degeneration of the somatic embryos, embryos at stages 2 to 4 from cell line 3:10 were isolated and cultured individually. The isolated embryos degenerated in a similar way as those embedded in embryogenic tissue, suggesting that the degeneration of somatic embryos was probably not induced by external factors. However, this does not rule out that the high frequency of degeneration was caused by a lack of stimulatory signals from the megagametophyte.

### Initiation of embryogenic tissue

The initiation step of embryogenic tissue in *Pinus* has so far only been briefly described (Finer *et al.* 1989; Becwar *et al.* 1990; Liao and Amerson 1995; Pullman *et al.* 2003; Lara-Chavez *et al.* 2011). Usually, whole megagametophytes, containing zygotic embryos at the stage of cleavage polyembryony, are used as a primary explant for initiating embryogenic cultures. One or more zygotic embryos protrude from the megagametophyte at the micropylar end, and somatic embryos start to form from this/these zygotic embryo(s). The embryo(s) will initially multiply and eventually form proliferating embryogenic tissue.

**Fig. 5.**
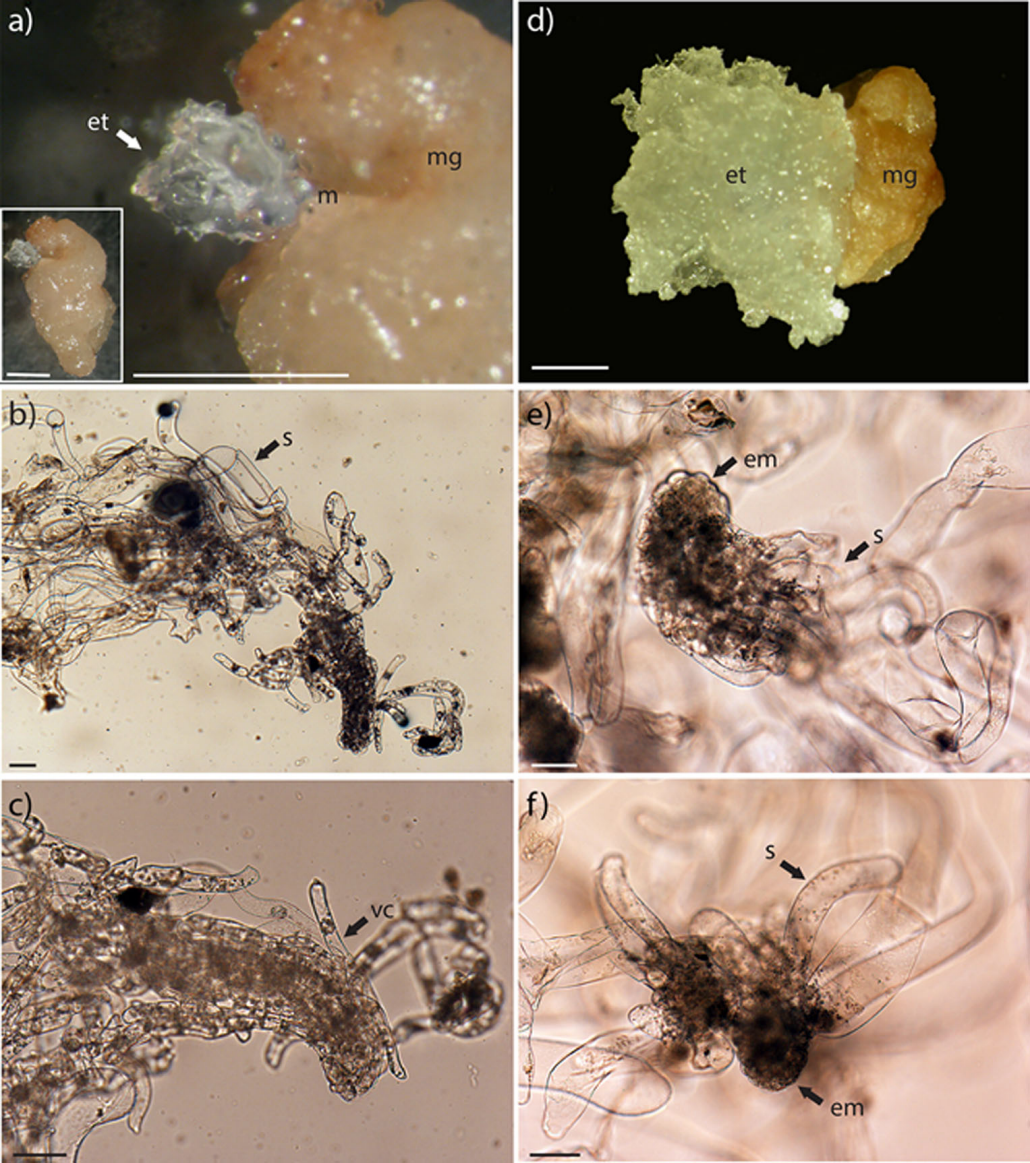
Initiation of embryogenic tissue from zygotic embryos. Isolated megagametophytes were incubated on proliferation medium. Embryogenic tissue at different stages was examined under a light microscope. (*a*) A megagametophyte with protruding embryogenic tissue at the micropylar end. The whole megagametophyte is shown in the inserted picture. (*b*, *c*) Higher magnification of the protruding embryogenic tissue shown in (*a*). Note the degenerating embryo in which elongated, vacuolated cells differentiate from the embryonal mass. (*d*) A megagametophyte with proliferating embryogenic tissue. (*e*, *f*) Higher magnification of the embryogenic tissue shown in (*d*). Note the early embryos consisting of a globular embryonal mass and suspensor cells. *et* embryogenic tissue, *em* embryonal mass, *m* micropyle, *mg* megagametophyte, *s* suspensor cells, *vc* vacuolated cells. *Bars* 1 mm (*a*, *d*) and 100 μm (*b*, *c*, *e*, *f*).

In order to elucidate when different degeneration patterns (i and ii) are established, we analyzed embryogenic tissues during the initiation phase (Fig. 5). Embryogenic tissue protruded at the micropylar end (Fig. 5 *a*). The protruding embryogenic tissue was composed of a degenerating embryo in which elongated, vacuolated cells differentiated from the embryonal mass (Fig. 5 *b*, *c*). When proliferation had started (Fig. 5 *d*), the embryogenic tissue consisted of several early embryos with globular embryonal masses and suspensor cells (Fig. 5 *e*, *f*). At the initial stage, in the protruding embryogenic tissue (Fig. S2a), dead cells were detected both in the suspensor and in the elongated, vacuolated cells (Fig. S2d). When the embryogenic tissue had increased in size (Fig. S2b), it consisted of meristematic cells and elongated, vacuolated cells and only a few dead cells were detected (Fig. S2e). To start with, the proliferating embryogenic tissue consisted of embryogenic cell aggregates (stage 1a) and early embryos (Fig. S2c). A high proportion of cells in the stage 1a aggregates (Fig. S2f), as well as in the early embryos (Fig. S2g), were dead. Successively, all the early embryos degenerated and proliferating embryogenic cultures consisting of embryogenic cell aggregates were established.

Our observations show that the zygotic embryo that protruded from the megagametophyte, initially degenerated before the formation of new embryogenic cell aggregates. This indicates that the cleavage process did not continue after initiation of embryogenic tissue. Furthermore, when the embryogenic tissue had started to proliferate, new early embryos differentiated, which subsequently degenerated. Interestingly, the embryos degenerated in a similar way as somatic embryos degenerating according to degeneration pattern (ii). Based on our results, we suggest that the continuous loop of embryo degeneration and differentiation of new embryos started already during the initiation of embryogenic tissue. The frequency of embryogenic cell lines of Scots pine giving rise to normal cotyledonary embryos is very low (normal cotyledonary embryos were obtained from 11 out of 325 cell lines; Burg *et al.* 2007; von Arnold, unpublished). Since the analyses of the initially protruding embryogenic tissue were destructive, it was not possible to determine if the differentiation pattern during the initiation phase differs between cell lines giving rise to normal and abnormal cotyledonary embryos. However, it seems that there is a high risk that embryogenic cultures established from immature zygotic embryos will be abnormal and give rise to abnormal cotyledonary embryos.

**Fig. 6.**
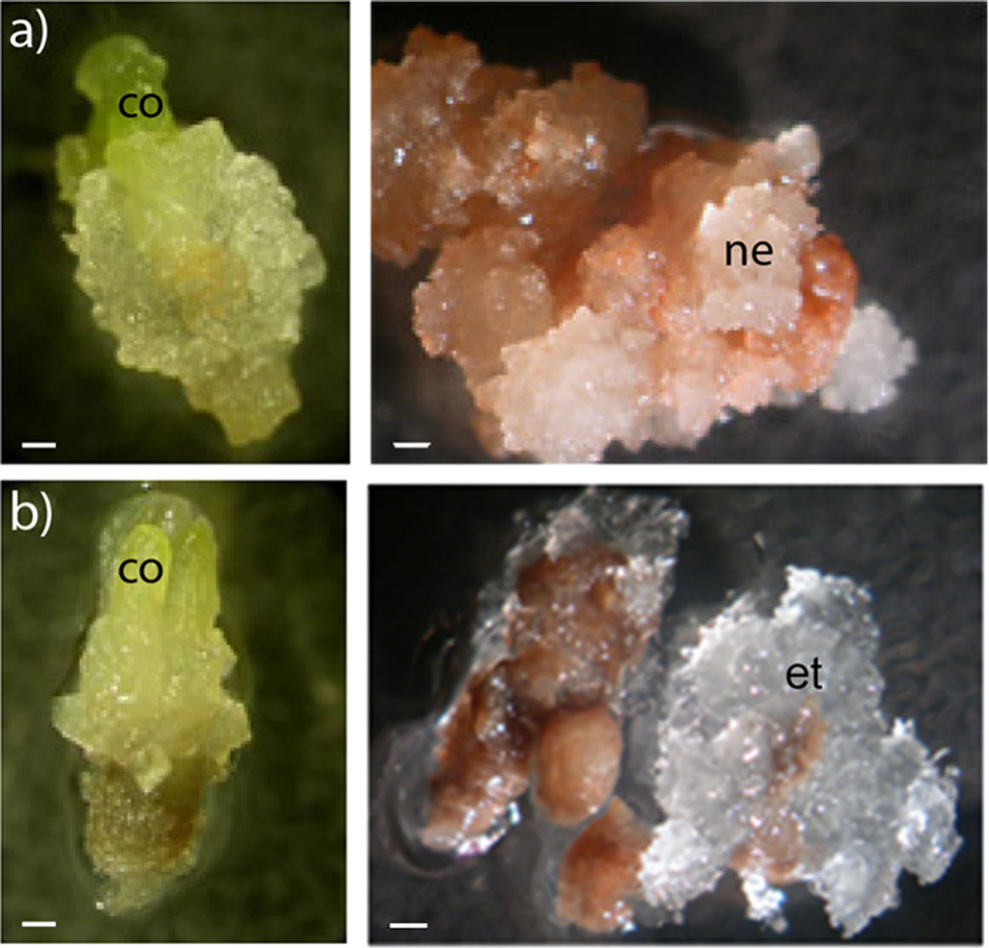
Initiation of embryogenic tissue from cotyledonary somatic embryos. Isolated cotyledonary embryos from cell lines 12:12 and 3:10 were incubated on proliferation medium containing TSA for 4 wk. (*a*) Differentiation of non-embryogenic callus from a cotyledonary embryo from cell line 12:12, after 2 wk (*left*) and after 4 wk (*right*). (*b*) Differentiation of embryogenic tissue from a cotyledonary embryo from cell line 3:10, after 2 wk (*left*) and after 4 wk (*right*). *co* cotyledons, *et* embryogenic tissue, *ne* non-embryogenic tissue. *Bars* 250 μm.

We further asked if a similar degeneration pattern would occur if embryogenic tissue was initiated from mature zygotic embryos. Embryogenic cultures in Scots pine have up to now mainly been established from immature zygotic embryos at the stage of cleavage polyembryony (Keinonen-Mättälä *et al.* 1996; Häggman *et al.* 1999; Burg *et al.* 2007; Lelu-Walter *et al.* 1999; Abrahamsson, unpublished). We have previously shown that treatment with TSA can stimulate the initiation of embryogenic tissue in germinated embryos of Norway spruce (Uddenberg *et al.* 2011). In order to test if treatment with TSA could affect the initiation of embryogenic tissue in Scots pine, cotyledonary zygotic and somatic embryos of cell lines 12:12 and 3:10 were screened for their potential to initiate embryogenic tissue after treatment with TSA (Fig. 6). No embryogenic tissue was formed on embryos not treated with TSA. Neither was embryogenic tissue induced, after TSA treatment, in cotyledonary zygotic embryos or somatic embryos of cell line 12:12 (Fig. 6 *a*). However, embryogenic tissue was initiated from approximately 70% of the cotyledonary embryos from cell line 3:10 after treatment with 10 μM TSA (Fig. 6 *b*). The newly initiated embryogenic cultures showed the same developmental and degeneration pattern as the “mother line”. In Norway spruce, treatment with TSA could maintain the embryogenic potential, although the treatment could not regain the embryogenic potential once it had been lost (Uddenberg *et al.* 2011). As expected, the embryogenic potential had been lost in cotyledonary zygotic embryos of Scots pine. The embryogenic potential had also been lost in cotyledonary somatic embryos with normal morphology, while the cotyledonary somatic embryos with abnormal morphology still retained the embryogenic potential. However, it remains unclear if embryogenic cell lines with degeneration patter (ii) would be initiated from cotyledonary embryos with normal morphology.

## Conclusion

A large proportion of early and late embryos in embryogenic cell lines of Scots pine degenerate. In a cell line giving rise to normal cotyledonary embryos, the degenerating embryos were eliminated similar to subordinate embryos in the seed. In contrast, in a cell line giving rise to abnormal cotyledonary embryos, the degeneration of the embryos resulted in a continuous loop of embryo degeneration and differentiation of new embryos. We suggest that the development of cotyledonary embryos with abnormal morphology in Scots pine was a consequence of the type of degeneration pattern of early and late embryos. Furthermore, the continuous loop of embryo degeneration and differentiation was established already during the initiation phase. Based on our results, we conclude that there is a high risk that embryogenic cultures of Scots pine, and probably embryogenic cultures of most species belonging to *Pinus*, established from zygotic embryos at the stage of cleavage polyembryony will be abnormal. Therefore, in order to avoid using immature zygotic embryos as the primary explant when initiating embryogenic cultures in *Pinus* species, further research is required for elucidating how to regain embryogenic potential in vegetative tissues.

## Electronic supplementary material

Table S1 (DOCX 21 kb).

Fig. S1 (DOCX 2081 kb).

Fig. S2 (DOCX 1798 kb).
