# Supplementary material for: Degeneration pattern in somatic embryos of Pinus sylvestris L
Source: In Vitro Cell Dev Biol Plant. 2017 Jan 26;53(2):86–96. doi: 10.1007/s11627-016-9797-y (PMC5423931; doi:10.1007/s11627-016-9797-y)
Supplement: Supplementary file 1 — (DOCX 21 kb) [file 11627_2016_9797_MOESM1_ESM.docx]

**Table S1**. Embryo developmental stages after four to six weeks on maturation medium

| **Time point** | **Proportion of embryos (%)** | | | | | |
| --- | --- | --- | --- | --- | --- | --- |
|  | stage 2 | stage 3 | stage 4 | stage 5 | stage 6 | stage 7 |
| 4w ABA | 50.0±1.0 | 31.5±0.5 | 16.5±0.5 | 1.5±0.5 | 0.0 | 0.0 |
| 6w ABA | 36.5±14.5 | 29.0±11.0 | 23.5±5.5 | 7.5±1.5 | 2.5±0.5 | 0.8±0.5 |

The proportion of embryos at different developmental stages in cell line 12:12 was estimated after four and six weeks on maturation medium containing ABA. Randomly selected samples from 12 biological replicates, in total 554 embryos, were classified according to developmental stage 2 to 7. The presented data give the proportion of embryos at each developmental stage ± SE.
