# Supplementary material for: Degeneration pattern in somatic embryos of Pinus sylvestris L
Source: In Vitro Cell Dev Biol Plant. 2017 Jan 26;53(2):86–96. doi: 10.1007/s11627-016-9797-y (PMC5423931; doi:10.1007/s11627-016-9797-y)
Supplement: Supplementary file 2 — (DOCX 2081 kb) [file 11627_2016_9797_MOESM2_ESM.docx]

**Figure S1.** Presence of a protoderm and a cuticularized layer on the surface of the embryonal mass. (*a*) A longitudinal section of an embryo, at stage 3 from cell line 3:10, which had developed a lobe. Note that the smooth surface of the embryonal mass is delineated by a protoderm, denoted by *arrows*. The suspensor cells were destroyed during fixation and embedding. (*b-f*) Embryos from cell line 3:10 stained with Oil Red O. (*b*) Embryo at stage 2. Note the strong positive staining of the embryonal mass. (*c*) Embryo at stage 3. Note the strong positive staining of the embryonal mass. (*d*) Embryo at developmental stage 3/4 carrying supernumerary suspensor cells. Note the strong positive staining of the embryonal mass. (*e*) An embryo that had disintegrated according to degeneration pattern (i) (degeneration pattern (i) is shown in Figure 3f). Note the disintegration of the embryonal mass and the vacuolization of the cells in the embryonal mass. Also note the scattered positive staining on the “former embryonal mass” indicating that the cuticularized layer has been partly degraded. (*f*) Embryo at developmental stage 3/4 that had degenerated into a less organized structure according to degeneration pattern (ii) (degeneration pattern (ii) is shown in Figure 3h). Note the strong positive staining of the embryonal mass and the elongated vacuolated cells, denoted by *arrow*, that differentiate from the embryonal mass. *em* embryonal mass, *lo* lobe, *s* suspensor cells, *vc* vacuolated cells. *Bars* 100 µm.

**Figure S1.**

**
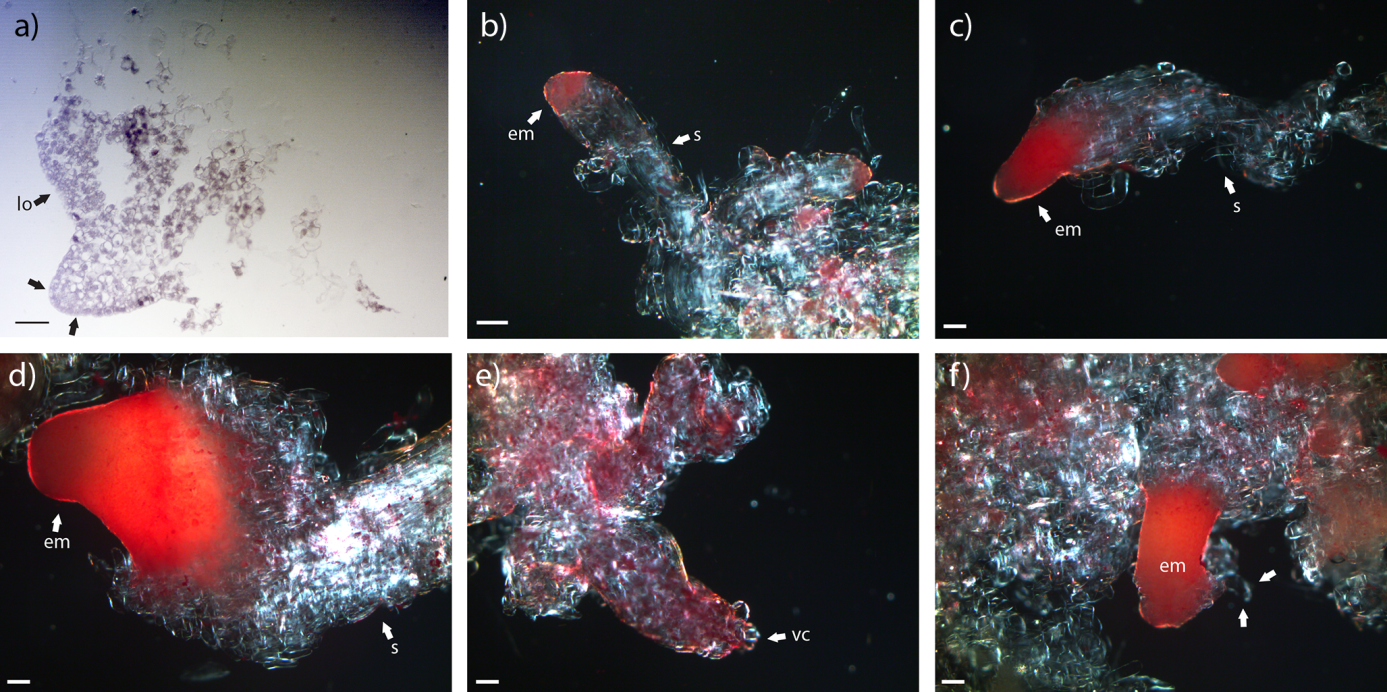
**
