# Supplementary material for: Degeneration pattern in somatic embryos of Pinus sylvestris L
Source: In Vitro Cell Dev Biol Plant. 2017 Jan 26;53(2):86–96. doi: 10.1007/s11627-016-9797-y (PMC5423931; doi:10.1007/s11627-016-9797-y)
Supplement: Supplementary file 3 — (DOCX 1798 kb) [file 11627_2016_9797_MOESM3_ESM.docx]

**Figure S2.** Initiation of embryogenic tissue from zygotic embryos. Isolated megagametophytes were incubated on proliferation medium. Embryogenic tissue at different stages was stained with Sytox^®^ and observed under fluorescent microscope. Red fluorescence indicates dead cells. (*a*) A megagametophyte with protruding embryogenic tissue at the micropylar end. The whole megagametophyte is shown in the inserted picture. (*b*) A megagametophyte with slightly more embryogenic tissue than the megagametophyte in (*a*). The whole megagametophyte is shown in the inserted picture. (*c*) A megagametophyte with proliferating embryogenic tissue. (*d*) Higher magnification of the embryogenic tissue shown in (*a*). Note the degenerating embryo with dead cells in the embryonal mass and in the suspensor, indicated by the *squares*. (*e*) Higher magnification of the embryogenic tissue shown in (*b*). The embryogenic tissue consisted of meristematic cells and elongated vacuolated cells. Note the few dead cells indicated by the *square*. (*f, g*) Higher magnification of embryogenic tissue shown in (*c*). Note the high proportion of dead cells in the embryogenic tissue containing embryogenic cell aggregates and early embryos consisting of a globular embryonal mass and suspensor cells. *ea* embryogenic cell aggregates, *ee* early embryo, *et* embryogenic tissue, *em* embryonal mass, *m* micropyle, *mg* megagametophyte, *s* supensor cells, *vc* vacuolated cells. *Bars* 1 mm (*a-c*); 100 µm (*d-g*).

**Figure S2.**

**
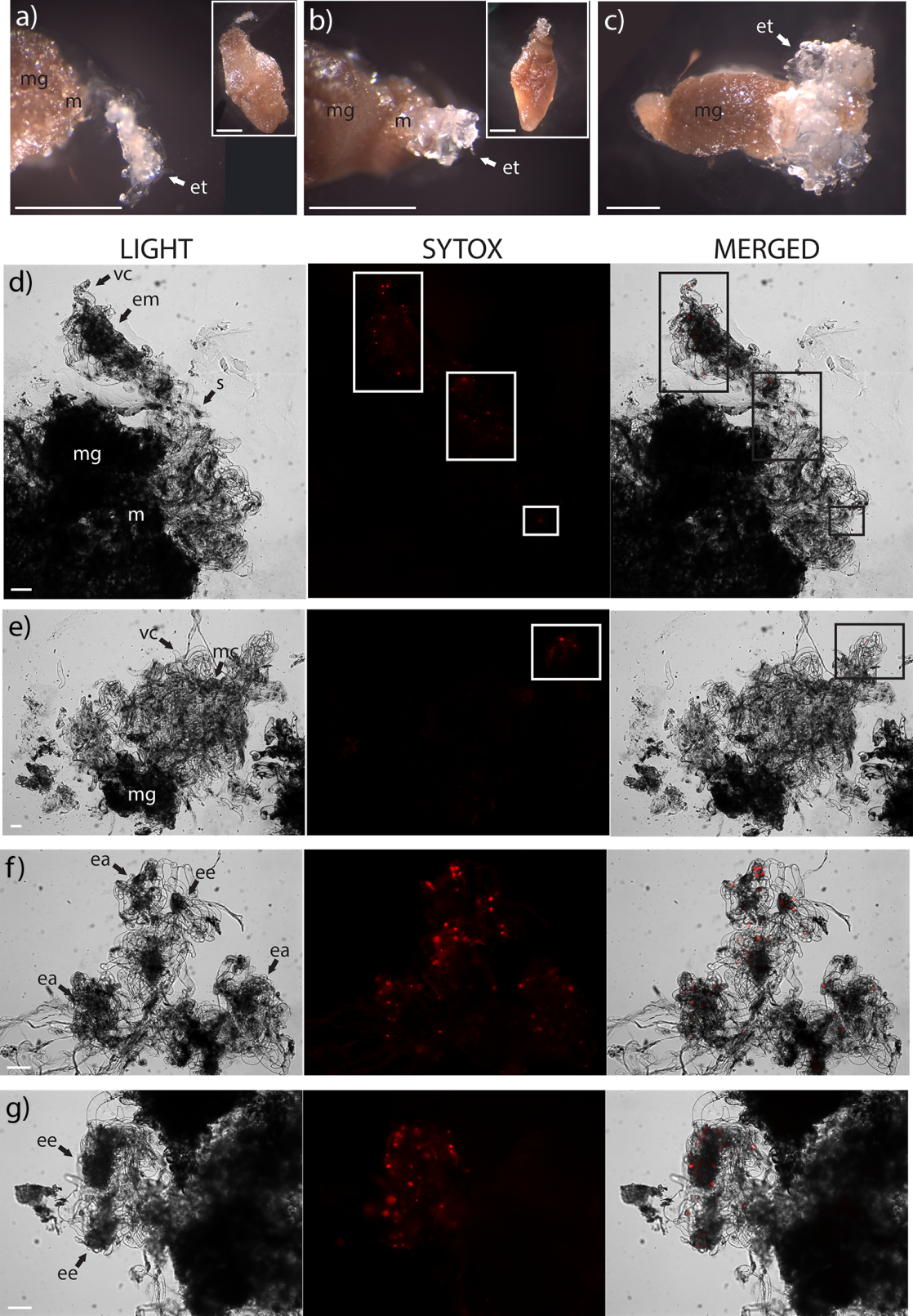
**
